# NF**κ**B and AP-1 Drive Human Myometrial IL8 Expression

**DOI:** 10.1155/2012/504952

**Published:** 2012-05-22

**Authors:** Shirin Khanjani, Vasso Terzidou, Mark R. Johnson, Phillip R. Bennett

**Affiliations:** ^1^Parturition Research Group, Institute of Reproductive and Developmental Biology, Imperial College London, London W12 0NN, UK; ^2^Academic Department of Obstetrics & Gynaecology, Chelsea and Westminster Hospital, Imperial College London, 369 Fulham Road, London SW10 9NH, UK

## Abstract

The uterine expression of the chemokine IL8 increases dramatically with the onset of labour both at term and preterm. The IL8 promoter contains binding sites for the transcription factors nuclear factor-kappa B (NF**κ**B), activator protein-1 (AP-1), and CCAAT/enhancer-binding protein (CEBP). In this study we investigated the roles of these transcription factors in IL1B regulation of the IL8 gene in human myometrium. Using chromatin immune precipitation (ChIP) assay, we showed that each of NF**κ**B, CEBP, and AP-1 binds to the IL8 promoter upon IL1B stimulation. To examine the relative importance of each site in IL8 gene expression, site-directed mutagenesis of each of these sites was performed. We found that the NF**κ**B site was essential for basal and IL1B-stimulated gene expression. Mutation of the AP-1 site reduced both basal and IL1B-stimulated expression but to a lesser extent. Mutation of the CEBP site had no effect upon basal expression but eliminated the IL1B response. Small interfering RNA (siRNA) silencing of NF**κ**B abolished the IL8 response to IL1B significantly; siRNA against AP-1 reduced it to a lesser extent whilst knockdown of CEBP enhanced the response. Our data confirms a central and essential role for NF**κ**B in regulation of IL8 in human myometrium.

## 1. Introduction

**Figure 1:**
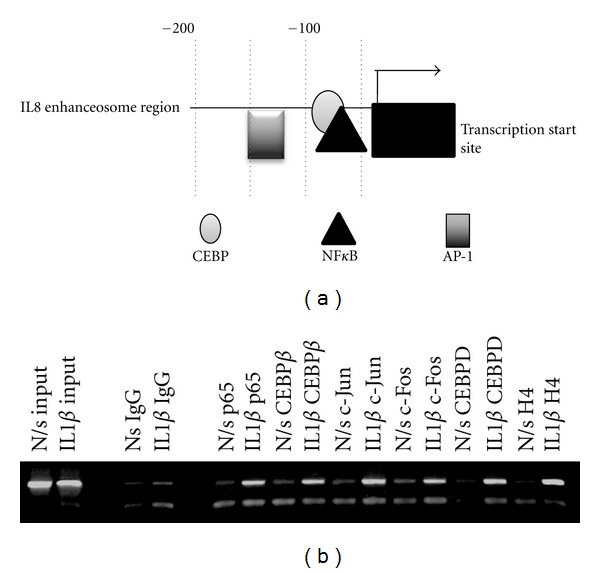
(a) Schematic of the IL8 promoter enhanceosome region. (b) ChIP analysis demonstrates the *in vivo* binding of NF*κ*Bp65, CEBP*β* and d, c-Jun, c-Fos, and H4 to the transcriptional enhanceosome of the IL8 promoter. ChIP assay using antibodies against NF*κ*Bp65 and CEBP*β* and -d, c-Jun, and c-Fos was performed in nonstimulated (N/s) and IL1B stimulated conditions. The immunoprecipitates were subjected to PCR analysis using primer pairs spanning the IL8 promoter transcriptional enhanceosome. The first two lanes are “input” lanes where no immunoprecipitation was performed prior to PCR. The second two lanes contained IgG antibody for immunoprecipitation (negative controls).

The onset of labour is associated with changes in gene expression in the myometrium consistent with activation of inflammatory mediators and leukocyte chemotaxis [1, 2]. The uterine expression of the chemokine IL8 increases dramatically with the onset of term and preterm labour [3, 4] and is thought to promote cervical remodelling [5, 6] and myometrial contractility by promoting neutrophil infiltration and activation [4, 7]. Myometrial IL8 expression is increased by stretch and the inflammatory cytokines in a MAPK- and NF*κ*B-dependent manner [8, 9]. Consistent with these observations, IL8 expression has been shown to be critically dependent on a region in its promoter, spanning nucleotides −1 to −133, which contains binding sites for the transcription factors AP-1, CEBP, and NF*κ*B [10–13]. The binding sites are in close proximity to each other and to the coding region of the gene, forming a transcriptional enhanceosome (Figure 1(a)). Many researchers have investigated the role and interaction of these transcription factors in regulating the IL8 gene and have shown different mechanisms of regulation depending on the cell type [13–21]. Studies in reproductive tissues show that NF*κ*B is important in IL1B-driven IL8 expression in amnion and myometrium [14], AP-1 is involved in the stretch-induced expression of IL8, OXTR, and COX2 in human myometrial and amnion cells [9, 22, 23], and CEBP has been shown to bind to two different regions of the IL8 promoter and interact with NF*κ*B to regulate the IL8 expression [24].

The marked inflammatory infiltration of the myometrium and cervix associated with human labour is driven by increased expression of IL8 and other chemokines. The transcription factors, NF*κ*B, CEBP, and AP-1, have all been implicated in IL8 expression in various tissues/cell types, but which actually regulate myometrial IL8 expression remains unclear. In this study, we have used IL1B stimulation to mimic labour in order to define the relative importance of NF*κ*B, CEBP, and AP-1 in myometrial IL8 expression.

## 2. Materials and Methods

### 2.1. Myometrial Cell Culture

Myometrial cells were extracted from biopsies taken at the time of elective caesarean section at term with the Ethics Committee approval and patient consent. None of the patients was in labour or had received uterotonics or tocolytics. Tissue was minced and digested for 45 min in DMEM with 1 mg/mL collagenase type IA and IX (Sigma-Aldrich Corp., St. Louis, MO, USA). Cells were centrifuged at 400 ×g for 10 min and grown in DMEM with 10% fetal calf serum, L-glutamine, and penicillin-streptomycin (37°C and 5% CO_2_). Cells were serum starved for 16 h before treatment with 1 ng/mL IL1B (R&D Systems, Inc., Minneapolis, MN, USA). Cells were used at passage three for all experiments.

### 2.2. Western Blotting

To obtain whole cell lysates, cells were lysed for 20 min on ice in radioimmunoprecipitation assay buffer (1% NP-40, 1% Triton, 1% sodium deoxycholate, 0.1% SDS, 150 mM NaCl, 10 mM Tris, PH 8.0, and 2 mM NaF). Protein concentrations were determined using detergent-compatible protein assay reagents (Bio-Rad Laboratories). Protein samples (50 *μ*g) were denatured by boiling for 5 min and run on a 10% SDS-PAGE for 60 min at 140 V, followed by a transfer to a Hybond ECL nitrocellulose membrane (Amersham Biosciensces). The membrane was blocked in 5% milk protein solution over night or for 1 hour, washed and hybridized with the primary antibody for 1 hour at room temperature in a fresh blocking buffer (1 × PBS, 1% milk protein and 0.1% Tween-20) containing antibodies for RELA and CEBP*β* (Santa Cruz Biochemicals, SC-8008 and SC-7962, resp.) or beta actin (Abcam, Ab6276). This process was repeated with the secondary antibody. The antibodies were used at 1 : 1000 dilution. Immunoreactivity was then visualised using a chemiluminescent substrate for HRP (ECL plus; Amersham Biosciensces).

### 2.3. Site-Directed Mutagenesis

The promoter region of the IL8 gene (−135/+46 bp) was amplified by polymerase chain reaction, and the fragment was ligated into the luciferase reporter plasmid pGL2-Basic (Promega, Southampton, UK) to give the wild-type construct. The mutation of individual base pairs in plasmid DNA was achieved using the QuikChange Site-Directed Mutagenesis Kit (Stratagene, UK) according to manufacturer's instructions. The mutagenic oligonucleotide primers were designed individually and are shown in Table 1.

### 2.4. Transient Transfection

Myocytes were grown in 24-well plates to 80% confluence. Transient transfections were performed using FuGene 6 transfection reagent (Roche, Indianapolis, IN, USA). Cytomegalovirus-*Renilla* vector (1/10th of reporter) was used to control for transfection efficiency and cell number. Luciferase reporter vectors containing either the wild-type IL8 promoter or the mNF*κ*B (mutation of NF*κ*B) or the mCEBP (mutation of CEBP) or the mAP-1 (mutation of AP-1) were transfected at 0.2 *μ*g/well. Cells were cultured for a total of 48 h (including 24 h of IL1B stimulation for half the experiment) followed by harvesting and analysis with a dual firefly/*Renilla* luciferase assay (Luclite, Packard Bell, and Coelenterazine CN Biosciences). Transfections were performed in triplicates. Luciferase/*Renilla* activity was measured 48 hours after transfection.

### 2.5. Chromatin Immunoprecipitation (ChIP)

DNA-protein complexes were crosslinked in situ with 1% formaldehyde. The cells were lysed, and chromatin was sheared into 200 to 1000 bp fragments by sonication. Antibodies recognizing the C-terminal of RELA, CEBP*β* (Santa Cruz Biochemicals, SC-8008 and SC-7962 resp.), and acetyl-histone H4 (Upstate cell signalling solutions, 06-866) were used (1 : 1000 dilution) for immunoprecipitation, and the chromatin fragments containing the crosslinked protein were purified by immunoabsorption and elution from protein A/G beads. The crosslinks were reversed, and the DNA was purified using a QIAquick nucleotide removal kit (QIAGEN Inc., Valencia, CA, USA). The DNA region crosslinked to the protein was determined by PCR analysis. PCR was performed using AmpliTaq Gold DNA polymerase (Applied Biosystems Ltd.). Pre-PCR cycle was 10 min at 95°C followed by 35 cycles of 95°C for 1 min, 56–60°C for 1 min, and 72°C for 1 min followed by final extension at 72°C for 10 min. The primers are shown in Table 2.

### 2.6. siRNA Transfection

ON-TARGETplus SMART pool human NF*κ*Bp65, CEBP*β*, CEBP*δ*, c-Fos, and c-Jun siRNAs (Dharmacon) were used. SiGLO (Dharmacon) was used as a positive control, giving a high transfection efficiency of approximately 90%, and ON-TARGETplus nontargeting pool (Dharmacon) was used as a negative control. The siRNAs were transfected using DharmaFECT 2 (Dharmacon) transfection reagent at a final concentration of 100 nM according to manufacturer's instructions. Briefly, cells were grown to 80% confluence. Two separate tubes were used for siRNA preparation. Tube one contained the siRNA diluted in phenol-red-free DMEM, and tube two contained the transfection reagent diluted in phenol-red-free DMEM. Each tube was incubated at room temperature for 5 min before mixing the contents of the two tubes. The mixture was incubated at room temperature for an additional 20 min. Prewarmed DMEM containing 10% FBS and 2 mM L-glutamine, but no antibiotics were then added to the mixture. The culture media was removed from the cells, and the cells were fed with the siRNA mixture. The media was changed to DMEM containing 10% FBS, 2 mM L-glutamine, and 100 U/mL penicillin-streptomycin 24 hours after the transfection. The siRNA transfections were performed at different time courses (12, 24, 48, and 72 h and 5, 6, and 7 days). The changes were of the same pattern at all time points, but maximal response was observed at 5 days. The cell viability was monitored closely before harvesting the cells for appropriate analysis.

### 2.7. Enzyme-Linked Immunosorbent Assay (ELISA)

At the end of incubation assays, 1 mL of medium was collected and immediately frozen at −80°C for analysis by IL8 ELISA kit (Biosource, KHC0081). The IL8 ELISA had a sensitivity of 5 pg/mL; the inter- and intra assay variations were 7.8% and 5.3%, respectively. ELISA data were normalised to cellular protein. Protein concentrations were determined using detergent-compatible protein assay reagents (Bio-Rad Laboratories).

### 2.8. Statistical Analysis

Data are expressed as mean ± SEM. Statistical analysis was performed by ANOVA with the level of significance defined as *P* < 0.05.

## 3. Results

### 3.1. IL1B Stimulates Binding of NF*κ*B, CEBP, and AP-1 to the IL8 Promoter

ChIP studies showed that IL1B induces the binding of NF*κ*Bp65, CEBP and AP-1 to the endogenous IL8 promoter. IL1B stimulated the recruitment of NF*κ*Bp65, CEBP*β*, and -*δ*, cFos, and cJun to their respective *cis*-elements in the transcriptional enhanceosome at 6 hrs (Figure 1(b)). This was associated with concomitant acetylation of H4 at that region of the promoter.

### 3.2. Site-Directed Mutagenesis Reveals an Essential Role for NF*κ*B in the Regulation of IL8 in Human Myometrium

**Figure 2:**
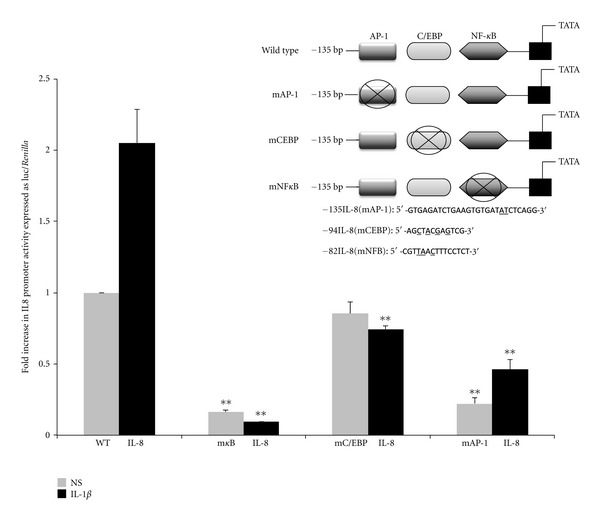
Representation of the IL8 promoter region and the mutations used in this study, as indicated by the underlined nucleotides. mAP-1: mutation of AP-1. mNF*κ*B: mutation of NF*κ*B and mCEBP: mutation of CEBP binding site. Functional effect of site-directed mutagenesis of transcription factor binding sites in the IL8 promoter in primary human myometrial cells. Data are presented as mean ± SEM (*n* = 4). An Anova test was performed with a Dunnett's multiple comparison post-test. **indicates a significant difference of *P* less than 0.01 compared to WT promoter.

A sequence extending from −135 to +46 which contains the transcription enhanceosome was used as a wild-type control. Site-directed mutagenesis was performed to make three further constructs with mutations for each of the NF*κ*B, CEBP, or AP-1 sites individually (Figure 2). Basal luciferase activity following transient transfection of the wild-type construct was significantly increased by 2-fold upon IL1B stimulation. In primary human myometrial cells, mutation of the NF*κ*B site reduced the unstimulated reporter activity to 10% of that of the wild type and obliterated the response to IL1B. Mutation of the CEBP site had no effect on basal promoter activity but did abolish the response to IL1B. Mutation of the AP-1 site decreased basal IL8 promoter activity although a response to IL1B stimulation was retained (Figure 2).

### 3.3. Small Interfering RNA (siRNA) Knockdown of NF*κ*B, CEBP, or AP-1 Demonstrates a Central Role for NF*κ*B in the Expression of IL8

**Figure 3:**
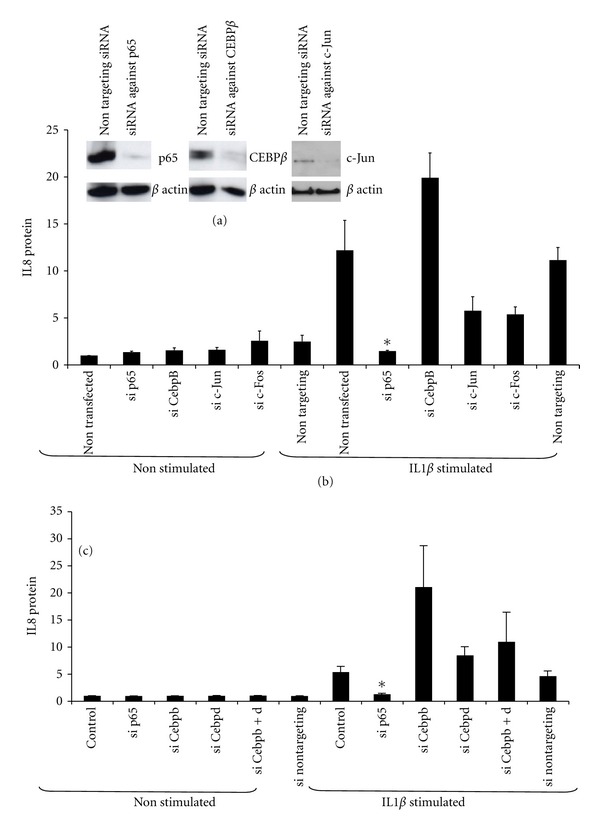
(a) siRNA knockdown of p65 (a), CEBP*β* (b), and c-Jun (c). (b) The effects of siRNA against NF*κ*Bp65, CEBP*β*, c-Jun, and c-Fos on IL8 protein production in a nonstimulated and IL1B-stimulated state. Data is normalised to the nontargeting control. Data are presented as mean ± SEM (*n* = 4). An ANOVA was performed with a Dunnett's multiple comparison post-test. *Indicates a significant difference of *P* less than 0.05. (c) The effects of siRNA against NF*κ*Bp65, CEBP*β*, and CEBPD on IL8 protein production in a nonstimulated and IL1B-stimulated state. Data is normalised to the non-targeting control. Data are presented as mean ± SEM (*n* = 4). An ANOVA was performed with a Dunnett's multiple comparison post-test. *Indicates a significant difference of *P* less than 0.05.

Knockdown mediated by siRNA against NF*κ*Bp65, CEBP*β*, c-Jun, and c-Fos was performed with protein expression studies showing highly-specific and better than 95% gene silencing (Figure 3(a)). The effect of siRNA knockdowns against NF*κ*Bp65, CEBP*β*, c-Jun, and c-Fos was measured on IL8 protein production and its response to IL1B stimulation (for 24 h) using ELISA.

Silencing of each individual transcription factor had no effect on basal IL8 protein release into the culture medium, measured by ELISA. Knock-down NF*κ*Bp65 abolished the response to IL1B significantly. Knockdown of CEBP*β* increased the response to IL1B by 1.5-fold whilst c-Jun and c-Fos knockdown reduced the response by half (Figure 3(b)). Since CEBP*δ* may also bind to the IL8 promoter (see Figure 1), we repeated the experiment using siRNA knockdown of CEBP*δ* which also increased the response to IL1B by 1.5-fold as with knockdown of CEBP*β* (Figure 3(c)).

## 4. Discussion

NF*κ*B, CEBP, and AP-1 are important transcription factor families that are involved in immune and inflammatory functions as well as in cell growth and differentiation. NF*κ*B, CEBP, and AP-1 are each expressed in human myometrium, and their expression is increased in response to the proinflammatory cytokine, IL1B. Labour-associated genes such as PGHS-2, OTR, IL8, IL6, and TNF-*α* have been shown to be regulated by either one or a combination of theses transcription factors [14, 16, 20, 21, 24–29].

Targeting activators of inflammation has been proposed as a strategy for prevention or delay of preterm birth and to improve outcome in preterm neonates. Condon et al. have demonstrated a central role for NF*κ*B in the onset of term labour in the mouse, and that inhibition of NF*κ*B can delay parturition [30]. Drugs that act through inhibition of NF*κ*B are available. For example, sulfasalazine is a synthetic anti-inflammatory drug comprising 5-aminosalicylic acid (5-ASA), linked to sulfapyridine, which is routinely used for the treatment of inflammatory bowel disease and rheumatoid arthritis. This has been shown to inhibit labour-associated gene expression in cell culture models [31, 32], although there are currently no animal or clinical studies. In a mouse model of inflammation-induced preterm labour the cyclopentenone prostaglandin J2, which acts via inhibition of NF*κ*B, both delays preterm birth and improves pup survival [33]. Glucocorticoids such as dexamethasone or prednisolone have well-established anti-inflammatory and immunosuppressive activities through their inhibitory effects on AP-1 and NF*κ*B pathways [34] although there is no evidence that these drugs will delay or prevent preterm birth. Progesterone, which, when used clinically, can reduce the risk of preterm labour, has been shown to repress IL1B-induced expression of NF*κ*B regulated genes in human amnion and myometrium [35, 36].

In this study, we examined the role of NF*κ*B, CEBP, and AP-1 in regulation of IL8 expression in human myometrium and used IL1B to mimic the effects of inflammation seen with human labour. We used ChIP to show that IL1B induces the binding of all three transcription factors to the IL8 promoter. This is consistent with the observations of Soloff et al. that IL1B increases both NF*κ*Bp65 and CEBP*β* binding to the endogenous IL8 promoter in myometrial cells [24]. Studies in other cell types have shown different patterns of binding. For example in a study in human conjunctiva epithelial cells ChIP showed no binding of NF*κ*B, CEBP, or AP-1 alone, but when immunoprecipitation was performed for both NF*κ*B and CEBP simultaneously, ChIP showed binding of both to the IL8 promoter [37]. Conversely treatment of human airway smooth muscle cells with tryptase, which mimics inflammation, induced the binding of all three transcription factors to the promoter [38].

Since we found that, in human myometrial cells all three transcription factors bound to the IL8 promoter, we considered two approaches to identify the relative importance of each transcription factor in IL8 expression. First we used site-directed mutagenesis to alter the binding sites for AP1, CEBP, and NF*κ*B. We found that mutating both the NF*κ*B and AP-1 sites markedly reduced both basal and stimulated IL8 promoter activity. However, mutating the CEBP site did not change the basal promoter activity but did completely inhibit the IL1B-induced increase in promoter activity. These data suggest that the NF*κ*B and AP-1 binding sites are essential for both basal and IL1B-stimulated IL8 expression. In contrast, it appeared that CEBP binding sites are not important for basal promoter activity but are essential for the IL1B-induced increase in promoter activity. Using a similar approach in human amnion and cervical epithelial cells, Elliott et al. found similar results for the NF*κ*B binding site but found no effect of mutating the CEBP or AP-1 binding sites on the IL8 promoter activity [14]. However, site-directed mutagenesis of DNA binding sites gives limited information about the role and importance of transcription factors, particularly where, as in the IL8 promoter, those sites are close to each other or overlap. Therefore in an alternative approach we used the more novel technique of siRNA against AP-1, CEBP, and NF*κ*B to knock down the expression of their endogenous proteins. The data generated by siRNA knockdown is probably the most reliable in terms of the role of individual transcription factors since it is highly specific. In addition, the readout is activity of the endogenous promoter, not a transfected construct. In contrast to the finding from the site-directed mutation studies, basal IL8 levels were not reduced by knockdown of any of the transcription factors. This may be because although we achieved 90% knockdown, this was not complete. However, knockdown of p65 did completely inhibit the IL1B-stimulated increase in IL8 levels. The effects of c-Jun and c-Fos knockdown were less marked but still inhibitory. CEBP knockdown did not inhibit the IL1B-induced increase in IL8 expression and actually tended to increase it. In some cell types CEBP has been shown to inhibit the action of NF*κ*B in regulation of IL8. Our knock-out data suggests that this phenomenon may apply in myometrium. Certainly our data does not point to a central positive role for CEBP in driving IL8 expression.

Our data show that, in human myometrial cells, NF*κ*B is essential for IL8 expression; AP-1 plays a less important but still stimulatory role, while the role of CEBP is less clear. The site-directed mutagenesis studies suggested a positive role in IL8 expression; however, the NF*κ*B and CEBP binding sites are close together, and it remains possible that mutation of the CEBP site may have interfered with NF*κ*B binding or function. The probably more reliable data from siRNA would suggest that pharmacological inhibition of CEBP might enhance IL8 expression and may therefore not be beneficial. The dominant role played by NF*κ*B in IL8 expression confirms that it is an attractive therapeutic target for the prevention of preterm labour.

## 5. Conclusion

These data show that NF*κ*B is essential for IL8 myometrial expression, AP-1 plays a less important but still stimulatory role, while the role of CEBP is less clear. These results provide further support for the notion that NF*κ*B represents an attractive therapeutic target for the prevention of preterm labour.

## Figures and Tables

**Table 1:** Primers used in site-directed mutagenesis.

Construct	Forward primer sequence	Reverse primer sequence
IL8 mAP-1	5′-GATCTGAAGTGTGATATCTCAGGTTTGCCCTGAGGG-3′	5′-CCCTCAGGGCAAACCTGAGATATCACACTTCAGATC-3′
IL8 m*κ*B	5′-GGGCCATCAGTTGCAAATCGTTAACTTTCCTCTGAC-3′	5′-GTCAGAGGAAAGTTAACGATTTGCAACTGATGGCCC-3′
IL8 mCEBP	5′-GGGCCATCAGCTACGAGTCGTGGAATTTCCTCTGAC-3′	5′-GTCAGAGGAAATTCCACGACTCGTAGCTGATGGCCC-3′

**Table 2:** IL8 ChIP primer covering bps −177 to +22.

Forward primer sequence	Reverse primer sequence
5′-GAAAACTTTCGTCATACTCCG-3′	3′-GAAAGTTTGTGCCTTATGGAG-5′
